# Why do young men not seek help for affective mental health issues? A systematic review of perceived barriers and facilitators among adolescent boys and young men

**DOI:** 10.1007/s00787-024-02520-9

**Published:** 2024-07-14

**Authors:** Ayesha Sheikh, Chloe Payne-Cook, Stephen Lisk, Ben Carter, June S. L. Brown

**Affiliations:** 1 Anna Freud, 4-8 Rodney Street, London, N1 9JH UK; 2 Department for Health, University of Bath, Bath, UK; 3 Department of Psychology, Institute of Psychiatry, Psychology and Neuroscience, King’s College London, London, UK; 4 Department of Biostatistics and Health Informatics, Institute of Psychiatry, Psychology and Neuroscience, King’s College London, London, UK

**Keywords:** Adolescent boys, Young men, Help-seeking, Barriers, Facilitators

## Abstract

Men are less likely to seek help for their mental health than women, but less is known about the specific patterns of help-seeking in adolescent boys and young men. This is concerning as adolescent boys and young men have high suicide rates but a low take-up of services. It is therefore of particular importance that the access needs of this group are understood. This review sought to identify the barriers and facilitators faced by adolescent boys and young men in help-seeking for affective mental health disorders. A search of the PubMed, APA PsycInfo, and Cochrane databases identified 3961 articles, of which 12 met the inclusion criteria. Six of the studies were qualitative, five were quantitative and one used mixed methods. Two authors independently extracted data and assessed the quality of the articles. Five key themes were identified, including the impact of social norms, with the subthemes of conformity to masculine norms and self-stigma, limited availability of information about mental health, and ‘male-friendly’ mental health literacy campaigns. Other themes referred to the help-seeking preferences of adolescent boys and young men, in terms of informal or formal and online or offline help-seeking. Some of the factors were well-researched (e.g., conformity to masculine norms as a barrier) whereas other factors (e.g., self-compassion as a facilitator) were less researched. These barriers and facilitators need to be considered in the development of future strategies to improve the help-seeking behaviour of adolescent boys and young men.

**Supplementary Information:**

The online version contains supplementary material available at 10.1007/s00787-024-02520-9.

## Introduction

The mental health needs of young people have been increasing over recent years, with nearly 17% of young people aged 5–16 reported to have experienced a mental health problem in 2022, up from 11% in 2017 [1]. The increase for 17–19-year-olds has been starker, with 25% likely to have had a mental health problem in 2022, compared to 10% in 2017 [1]. A recent survey by the Mental Health Foundation [2] found that 89% of young people aged between 18 and 24 have been affected by anxiety in their daily life. The needs of adolescents are reflected in referrals to the UK’s National Health Service (NHS) Child and Adolescent Mental Health Services (CAMHS), with 11–15-year-olds making up the majority of referrals for young people in 2015 [3].

Research has previously indicated that nearly half (48.4%) of mental health problems occur before age 18, with 62.5% occurring before age 25, and the peak age of onset being 14.5 years [4]. This highlights the importance of addressing these issues during adolescence and early adulthood and researching help-seeking during this period of development. Adolescent boys and young men are at particular risk of poor mental health outcomes: in university students, the suicide rate for male students is significantly higher than that for female students, and this rate increases in first year undergraduate men [5]. However, this increased need does not lead to increased use of support services by young men. Adolescent boys and young men are underrepresented in referrals to NHS psychological therapy services in the UK, with recent statistics showing that male-identifying 18–25-year-olds accounted for less than 30% of referrals [3, 6]. Previous research in Australia showed that only 13.2% of young men aged 16–24 with mental health needs had accessed mental health services in the 12 months prior [7]. More recently, evidence has shown that Australian men aged 18–25 had decreased service use during the first year of the COVID-19 pandemic, whereas young women in the same age group accessed services more, when both groups were compared to the previous year [8].

Adolescent boys and young men are evidently not seeking help when they need it, but there is a lack of research into why they are reluctant to do so. Studies on help-seeking behaviours often focus on youth in general, older men or clinical populations. For youth in general, Rickwood et al. [9] found that those who were more reluctant to seek help for mental health often viewed help-seeking as a negative process or saw mental health difficulties as something they could solve themselves. Gulliver et al. [10] discussed how stigma and a lack of mental health literacy were barriers to help-seeking for young people. Regarding older adult male populations, Addis and Mahalik [11] proposed that the traditional notions of masculinity such as “self-reliance, emotional control, and power” [11] (p. 12) mean that men feel unable to approach services, for fear of being ‘othered’ by their peers. It has also been suggested that men are simply less likely to acknowledge when they have a psychological problem, or do not see mood disorders such as depression as serious enough to warrant help-seeking, and therefore will not seek help as readily as women [12, 13].

To improve male help-seeking and engagement with interventions, it has been suggested that mental health services should utilise a male-friendly approach that uses non-stigmatising language [14, 15]. This idea was also raised in a review by Sagar-Ouriaghli et al. [16] which looked at behaviour change techniques for men of all ages. It was further suggested in this review that men could be better encouraged to seek help if it was demonstrated to fit in with traditional masculine norms of showing strength and responsibility [16]. Other behaviour change techniques were the use of male role models and the provision of information to improve mental health literacy and symptom recognition [16].

It is therefore important to understand barriers and facilitators to seeking help for affective mental health problems among adolescent boys and young men so that interventions and programmes can be developed that are accessible and effective for them. Encouraging help-seeking at the early stages of mental health difficulties could ensure that problems are addressed before they develop into more serious issues, such as severe depression or suicide. This review aimed to address the gap in the literature by identifying the perceived barriers and facilitators to help-seeking for mental health in adolescent boys and young men.

## Methods

This systematic review has been designed and reported in line with the PRISMA statement.

### Definitions of search terms

For the purposes of this review, barriers and facilitators were defined as any factors that may impact the decision of adolescent boys and young men to access help for mental health, therefore terms such as ‘hurdle’, ‘obstruction’, ‘support’, and ‘engagement’ were also included in the search. Mental health is described by the World Health Organization (WHO) [17] as “a state of mental well-being that enables people to cope with the stresses of life” [17] (para. 1), but for this review it also included the concepts of affective mental health disorders such as anxiety or depression. Broader terms like stress and wellbeing were also included in the search terms to be as comprehensive as possible and to capture any relevant studies that may not have referred to mental health specifically. Finally, the action of help-seeking was considered to be any form of seeking support, and searches included related terms such as ‘treatment-seeking’ and ‘help-seeking behaviour’. An example of the full search terms used can be seen in the appendix Supplementary Information 1.

### Study eligibility

Studies were included based on the following criteria:

1. Study samples were adolescent boys and young men aged 14–22 years (or mean age of sample aged between 14 and 22 years).
2. Male-only samples, or if mixed gender then only males were included in the data analysis.
3. Participants were seeking help for affective mental health concerns (e.g. depression, anxiety), or discussing intentions or likelihood of seeking help.
4. No more than 50% of participants in a study with a clinical diagnosis of an established mental health condition.
5. Randomised controlled trials (RCTs), cross-sectional, qualitative and mixed method studies.
6. English language publications.

Criteria 4 was selected on the basis that those with a diagnosis were more likely to be mental health service users who were already help-seekers. Studies were excluded if they involved participants with more severe mental health conditions or non-mood disorders such as psychosis, eating disorders or addiction. Institutional studies (i.e., wholly within inpatient or clinical settings) were also excluded. Additionally, studies were excluded if they did not come under Criteria 5 and were articles such as commentaries, editorials, opinion pieces or other systematic reviews. There were no restrictions on year of publication.

### Search strategy

From inception until 8th December 2023, the following databases were searched for potentially relevant studies: PubMed, APA PsycINFO and Cochrane. Each database was searched for concepts related to the three key terms of barriers and facilitators, mental health and help-seeking.

### Screening

Results from the searches were uploaded to the review management software Rayyan and duplicates were removed. The remaining studies were screened by title and abstract independently by CPC and AS. Any conflicts were resolved through discussion with the other authors. Full-text articles were retrieved and screened for the remaining studies by CPC and AS and the final decisions were made on study inclusion.

### Methods of quality assessment

Two methods of quality assessment were used to assess the included studies. The Critical Appraisal Skills Programme (CASP) checklist [18], which uses 10 items to assess various factors of each study, was used for the six qualitative studies and one mixed methods study. Studies were rated as either ‘valuable’ or ‘not valuable’, depending on if their contribution to existing literature and generalisability was discussed, along with directions for future research. For the remaining five quantitative studies, the Newcastle–Ottawa Quality Assessment Scale (NOS) for cross-sectional studies [19] was used. Studies were scored either ‘poor’, ‘fair’ or ‘good’ based on three sub-domains of selection, comparability, and outcome. The lowest domain score was used as the overall rating for each study.

### Data extraction, synthesis, and analysis

A data extraction table was created using the following categories: aims, methods, study population, sample size, interventions, outcomes, results, conclusions/implications, strengths and limitations.

Thematic analysis was conducted on the results sections of the six qualitative papers, and the qualitative results of the one mixed methods paper, according to the six principles set out by Braun and Clarke [20]. The results section of each paper was uploaded to the NVivo software to assist with generating initial codes and themes. CPC coded all the data line-by-line, and AS conducted the same process on just under 50% of the papers. Themes were discussed and refined by CPC and AS and confirmed with the other authors. A convergent integrated approach for mixed methods systematic reviews was then followed, according to JBI guidance [21], in which the results of the five quantitative papers were ‘qualitised’ by converting the extracted data into text. This then allowed for the data to be incorporated into the existing themes to create a narrative synthesis.

## Results

**Fig. 1:**
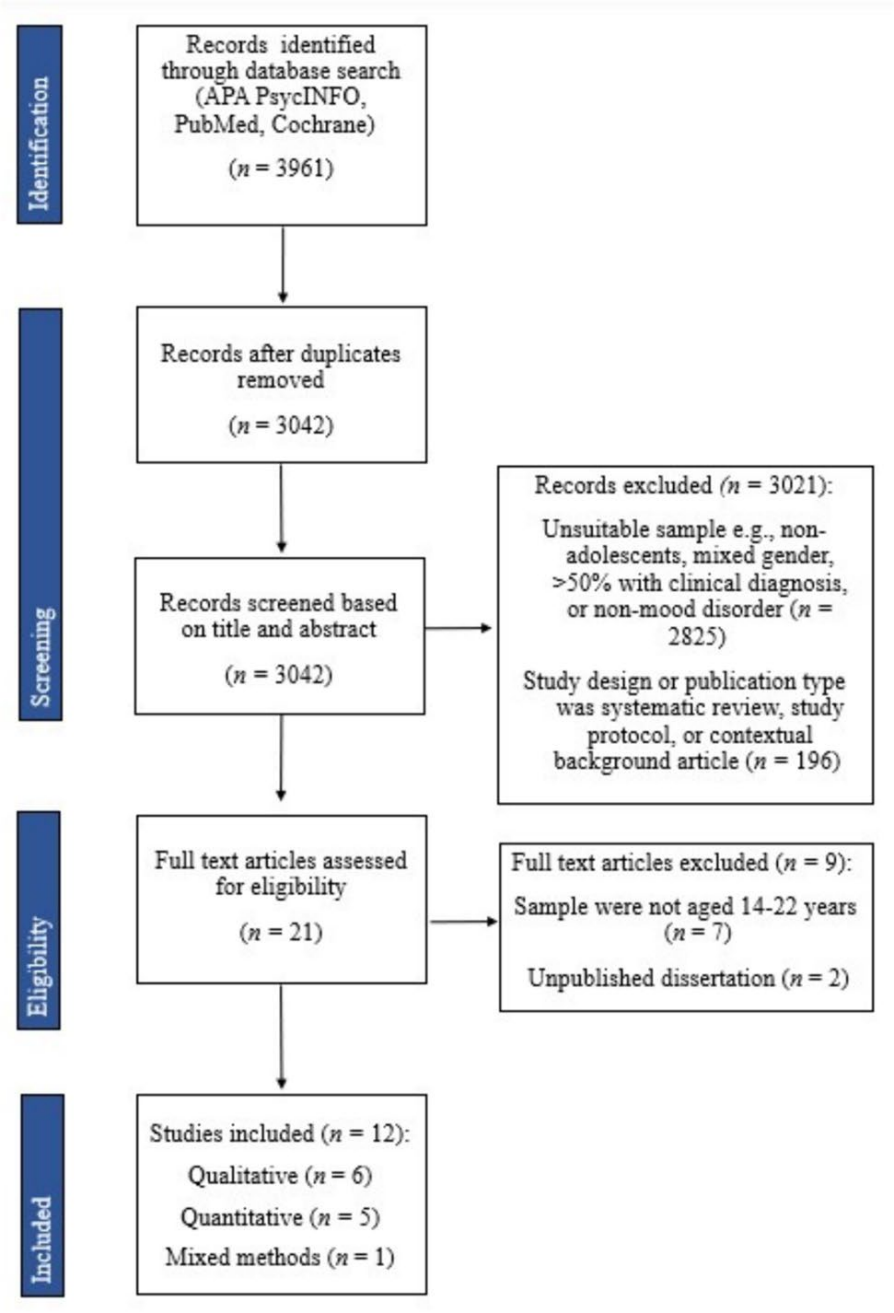
PRISMA flowchart of the study selection process

In total, 3961 articles were identified through database searching and 12 of these articles met the inclusion criteria. These 12 articles were therefore included in the review and provided information that could be extracted on the barriers and facilitators to help-seeking for mental health in adolescent boys and young men (Fig. 1).

### Types of included studies

Six of the studies included were conducted using qualitative methods, such as focus groups and semi-structured interviews, and five studies were conducted using quantitative methods, namely survey questionnaires. One included study used a mixed methods approach with both surveys and focus groups.

### Study characteristics

A table of study characteristics (Table 1) was created using the following categories: study type, study author(s) and year, age range and mean age, sample size, country, study context, study methods and aim, type of analysis, key findings, and study implications. The number of study participants included across the 12 included studies totalled 4842 (M = 403.5; SD = 727.82). The average participant age in each study in this review was within the inclusion criteria of 14–22 years.

**Table 1:** Characteristics of selected studies

Study author and year	Age range (Mean)	Sample size	Country	Context	Methods and aims	Analysis	Key findings	Implications
Qualitative studies								
Best, P., Gil-Rodriguez, E., Manktelow, R., & Taylor, B. J. (2016)	14–15 (n/a)	56	UK (N Ireland)	School	Photos used to generate focus group discussion about process of online help-seeking (HS)	Thematic analysis	Informal online HS from social networks was easier to access and more likely to be used, but concerns felt over anonymity – formal pathways were seen to be more trustworthy	Online technology can be used to facilitate formal HS for mental health (MH) in male adolescents, but they should also be taught how to filter out poor-quality or inaccurate information
Burke, L., John, M., & Hanna, P. (2022)	16–18 (n/a)	14	UK	School (sixth form)	1:1 semi-guided interviews to explore how informal relationships affect HS	Thematic analysis	Two key themes identified in informal relationships that affect HS: proximity to and familiarity with others, and navigating feared outcomes	Young males could benefit from having dedicated spaces to discuss MH, with input from male role models. Schools may be ideal setting for future interventions
Clark, L. H., Hudson, J. L., Dunstan, D. A., & Clark, G. I. (2018)	12–18 (15.17)	29	Australia	Clinical service, community and schools	Vignettes given in semi-structured interviews and focus groups to identify barriers and facilitators	Grounded theory	Barriers included stigma and masculine norms, and limited knowledge of anxiety. Facilitators were having accessible MH literacy programmes and a wide range of options for HS	Adolescent males need more options for accessing MH support and information, especially related to anxiety. Stepped/discrete approaches may encourage more HS
Lynch, L., Long, M., & Moorhead, A. (2018)	18–24 (FG = 21.3, Interview = 20.8)	17	Ireland	Youth service	Focus groups and interviews to explore barriers and solutions to professional HS	Thematic analysis	Four main barriers to HS: fear of medication, fear of homophobic responses from professionals, legacy of Catholic attitudes in Ireland, and genuine need for care	Services and professionals need to actively address barriers, and combine advertising, services and education. Young men should be respected and involved in creating strategies
Meechan, H., John, M., & Hanna, P. (2021)	16–18 (n/a)	10	UK	School	Interviews to examine how young Black males see MH systems in UK	Thematic analysis	For young Black men, formal support was seen as unfamiliar, unapproachable and discriminatory. Community attitudes may impact informal HS	Black male role models can encourage discussion of MH. More Black MH professionals needed to make service more representative and relevant
Sagar-Ouriaghli, I., Brown, J. S. L., Tailor, V., & Godfrey, E. (2020)	18–31 (21.89)	24	UK	University	Focus groups to identify approaches that affect and would improve HS	Thematic analysis	Key themes identified around HS, related to masculinity, intervention format preferences and knowledge around HS	Interventions could be made male-friendly by being male-only, using male role models and targeting stigma and attitudes
Quantitative studies								
Cole, B. P., & Ingram, P. B. (2020)	18–22 (n/a)	313	USA	University	Measures and vignette via survey, to assess gender role conflict, self-stigma and HS	SEM (structural equation modelling)	Gender role conflict increased avoidant behaviours and decreased use of social support. Increased self-stigma was linked to decreased informal and formal HS	Programmes should be developed to normalise depression and HS in this population, and interventions should address the impact of GRC and self-stigma
Heath, P. J., Brenner, R. E., Vogel, D. L., Lannin, D. G., & Strass, H. A. (2017)	18–30 (19.68)	284	USA	University	Measures via online survey to assess impact of self-compassion on barriers to HS	SEM (structural equation modelling)	Self-compassion (showing oneself kindness) was associated with fewer perceived barriers to HS, such as masculine norm adherence and self-stigma	Interventions should be developed that target self-compassion, as this could decrease some of the key barriers and therefore increase HS
Liddle, S. K., Robinson, L., Vella, S. A., & Deane, F. P. (2021)	12–18 (14.69)	1038	Australia	Community sports clubs	Measures via paper survey to identify subgroups of HS intentions	LPA (latent profile analysis)	Adolescents at high-risk of MH difficulties were less likely to seek help, especially from parents, and had lower family support	Interventions could target support from family and friends to increase HS in high-risk groups
Ramaeker, J., & Petrie, T. A. (2019)	n/a (Athletes = 20.03 Non-athletes = 21.40)	Athletes = 220 Non-athletes = 205	USA	University	Measures via survey to assess differences between athletes and non-athletes in attitudes to HS	MANOVA, multiple regression and path analysis	Athletes more strongly identified with certain masculine norms, but overall conformity to these was associated with more negative attitudes about HS in both groups	Public information campaigns and support networks may aid men’s formal and informal help-seeking. Male role models based in sport may also help to reduce stigma by sharing their own MH stories
van der Schyff, E. L., Amon, K. L., Ridout, B., Forsyth, R., & Campbell, A. J. (2023)	18–83 (21.37)	2515	Mainly Australia, UK, and USA	Online video gamers	Measures via online survey to assess confidence in HS in male gamers, and HS barriers	Frequency analysis and multiple regression analysis	Male gamers had concerns over confidentiality and trusting clinicians when seeking formal help. Reported embarrassment with HS, and low confidence in MH symptom recognition or understanding MH information	Strategies needed to overcome these specific barriers in male gamers. Normalising HS behaviour in a supportive, understanding, and non-judgmental environment may increase confidence in HS
Mixed method studies								
Ellis, L. A., Collin, P., Hurley, P. J., Davenport, T. A., Burns, J. M., & Hickie, I. B. (2013)	16–24 (18.5)	117	Australia	Variety – youth centres, universities, clinics, schools	Online survey and focus groups to explore attitudes to MH and technology use	Linear regression and thematic analysis	Young men prefer online self-help to other forms of HS, due to barriers such as notions of masculinity and negative attitudes towards professionals	Online MH services need to design interventions for young men that focus on changing behaviour and reducing stigma, and not just for increasing MH knowledge

Four of the studies included were based in the UK, three were based in Australia, three were based in the USA and one was based in Ireland. One study was Australian based but included an international sample, with participants mostly from the USA, Australia, and the UK. The majority of the studies included in this review (7) took place in a school setting or university setting. Three studies took place in community-based settings, including sports clubs and clinics. One study took place within a youth service, and one used an online sample of self-reported video gamers.

### Quality assessment of studies

The six qualitative and one mixed methods studies were all listed as ‘valuable’ according to the CASP criteria, although three of these studies did not adequately report on reflexivity. For the five quantitative studies assessed using the NOS, one study was rated as Good, whereas the other four were rated as Fair. This was mainly due to issues around comparability and how well studies controlled for confounding variables. However, all quantitative studies rated highly in terms of selection and outcome quality (see Tables 2 and 3 for further details).

**Table 2:** CASP checklist for qualitative research summary table

	Was there a clear statement of the aims of the research?	Is a qualitative methodology appropriate?	Was the research design appropriate to address the aims of the research?	Was the recruitment strategy appropriate to the aims of the research?	Was the data collected in a way that addressed the research issue?	Has the relationship between researcher and participants been adequately considered?	Have ethical issues been taken into consideration?	Was the data analysis sufficiently rigorous?	Is there a clear statement of findings?	How valuable is the research?
Best, P., Gil-Rodriguez, E., Manktelow, R., & Taylor, B. J. (2016)	Yes	Yes	Yes	Yes	Yes	No—no mention of position of researcher	Yes	Yes	Yes	Valuable
Burke, L., John, M., & Hanna, P. (2022)	Yes	Yes	Yes	Yes	Yes	Yes	Yes	Yes	Yes	Valuable
Clark, L. H., Hudson, J. L., Dunstan, D. A., & Clark, G. I. (2018)	Yes	Yes	Yes	Yes	Yes	No—no mention of position of researcher, especially related to gender	Yes	Yes	Yes	Valuable
Ellis, L. A., Collin, P., Hurley, P. J., Davenport, T. A., Burns, J. M., & Hickie, I. B. (2013)	Yes	Yes	Yes	Yes	Yes	No—no mention of position of researcher	Yes	Yes	Yes	Valuable
Lynch, L., Long, M., & Moorhead, A. (2018)	Yes	Yes	Yes	Yes	Yes	Yes	Yes	Yes	Yes	Valuable
Meechan, H., John, M., & Hanna, P. (2021)	Yes	Yes	Yes	Yes	Yes	Yes	Yes	Yes	Yes	Valuable
Sagar-Ouriaghli, I., Brown, J. S. L., Tailor, V., & Godfrey, E. (2020)	Yes	Yes	Yes	Yes	Yes	Yes	Yes	Yes	Yes	Valuable

Studies were graded as 'valuable' if:

- The researcher discusses the contribution the study makes to existing knowledge or understanding (e.g., do they consider the findings in relation to current practice or policy, or relevant research-based literature)

- They identify new areas where research is necessary

- The researchers have discussed whether or how the findings can be transferred to other populations or considered other ways the research may be used

**Table 3:** NOS for cross-sectional studies

	Selection (max. stars 5)				Comparability (max. stars 2)	Outcome (max. stars 3)		Sub-total assessment			Rating
	Representativeness	Sample size	Non-respondents	Ascertainment of exposure	Control of confounding variables	Assessment of outcome	Statistical test	Selection	Comparability	Outcome	
Cole, B. P., & Ingram, P. B. (2020)	*	*–*	*–*	**	*	*	*	Good	Fair	Good	Fair
Heath, P. J., Brenner, R. E., Vogel, D. L., Lannin, D. G., & Strass, H. A. (2017)	*	*	–	**	*	*	*	Good	Fair	Good	Fair
Liddle, S. K., Robinson, L., Vella, S. A., & Deane, F. P. (2021)	*	–	–	**	**	*	*	Good	Good	Good	Good
Ramaeker, J., & Petrie, T. A. (2019)	*	–	–	**	*	*	*	Good	Fair	Good	Fair
van der Schyff, E. L., Amon, K. L., Ridout, B., Forsyth, R., & Campbell, A. J. (2023)	*	–	–	**	*	*	*	*Good*	Fair	Good	Fair

Domain scores for cross-sectional studies: Selection = 0–1 (Poor); 2 (Fair); 3+ (Good); Comparability and Outcome = 0 (Poor); 1 (Fair); 2+ (Good). * = meets criteria for acceptability

This scale has been adapted from the Newcastle–Ottawa Quality Assessment Scale for cohort studies to provide quality assessment of cross-sectional studies [19]

In the Outcomes section of this scale, one star has been specifically assigned for self-reported outcomes, because the study measures help-seeking intentions or attitude towards help-seeking. Two stars would be awarded to any studies that measured actual help-seeking

### Themes identified

Analysis of the 12 included articles identified five distinct themes related to barriers, facilitators, and general trends in adolescent boys and young men’s help-seeking. These are highlighted in Table 4 and discussed in detail below.

**Table 4:** Categories of themes identified

	Themes	Subtheme
Barriers	The impact of social norms	Conformity to masculine norms
		Self-stigma
	Limited availability of information about mental health and help-seeking	
Facilitators	“Male-friendly” campaigns to promote interventions or mental health literacy	
	Self-compassion	
Other notable themes	Help-seeking preferences	Informal vs. formal help-seeking
		Online vs. offline help-seeking

### Theme one: the impact of social norms

#### Papers with this theme: 12 [22–33]

*The impact of social norms* was identified as a key barrier to help-seeking. This can be further organised into two subthemes: *conformity to masculine norms* and *self-stigma*.

### Subtheme one: conformity to masculine norms

All 12 papers identified *conformity to masculine social norms* (CMN) as a barrier to adolescent boys and young men’s help-seeking for mental health, often due to the stigmatisation of mental health difficulties being seen as a ‘weakness’ [22–33]. CMN can be defined as rigid adherence to stereotypical notions (both positive and negative) of masculinity posited by society [34, 35]. Adolescent boys and young men felt that seeking help was embarrassing as it contradicted traditional notions of masculinity, and that they should be able to deal with problems without professional help [26, 29, 32, 33]. CMN was therefore associated with more negative attitudes towards help-seeking, which can be seen by young men as a weak behaviour and, consequently, one to be avoided [29, 31]. For example, one participant in Clark et al.’s study [24] stated “Yeah, there’s a sort of stereotype of males…. if you are suffering from one of those [mental health problems] that you are weaker than everyone else” [24] (p. 230).

Cole and Ingram [25] examined the relationship between ‘gender role conflict’ (GRC) and help-seeking. GRC is defined by O’Neil [36] as “a psychological state in which socialized gender roles have negative consequences for the person or others” [36] (p. 362). Cole and Ingram found that GRC in young men led to increased avoidant behaviour and decreased use of social support for mental health concerns, however GRC did not have an impact on seeking professional psychological support. GRC was also found to be a key factor in the gendered differences of help-seeking, which is in parallel with the findings of Sagar-Ouriaghli et al. [32] around traditional masculine stereotypes.

Masculine norms were also found to be a specific barrier for young Black men in the UK in Meechan et al.’s [30] paper. This study focused on the intersections of race and masculinity on help-seeking behaviour and found that Black men felt social pressure to be strong and to cope independently. Interestingly, all participants in this study identified mental strength as a key traditional trait for Black men in the UK and understood seeking help for mental health in the “cultural context of generations of African Caribbean men needing to be strong to overcome racism” [30] (p. 7). Participants also discussed this in comparison to white males, noting that “the notion of masculinity and strength was more pronounced within Black male communities” [30] (p. 4). CMN may therefore present differently for white and non-white ethnic groups.

### Subtheme two: self-stigma

CMN is closely linked to the subtheme of *self-stigma*. All 12 papers acknowledged stigma or stigmatising attitudes towards mental health [22–33]. Cole and Ingram [25] define self-stigma as “the belief that one is inadequate or weak, if he wants to seek professional help” [25] (p. 442). Adolescent boys and young men may view help-seeking as a personal failure, as they have been unable to solve the problem themselves. The specific relationship between masculine norm adherence and self-stigma was highlighted by several studies, with men who endorse greater masculine norms experiencing higher levels of self-stigma [25, 27, 31]. Cole and Ingram [25] reported that men who experience higher levels of self-stigma are less likely to seek informal help from friends, family, and peers. They also found that these men were more likely to practice avoidant behaviours, which may help to explain comorbidity of substance abuse disorders in this age group [25].

Heath et al. [27] identified self-stigma as a barrier for adolescent boys and young men that is consistently associated with lower help seeking intentions. Heath et al. [27] used a self-stigma scale to measure the relationship between this and professional help-seeking in participants and found that self-stigma was positively related to self-disclosure risks. Meechan et al. [30] found that stigma within Black communities influenced young men’s identities, and that generational stigma was passed down through families and served as a barrier for help-seeking within this community, which is similar to Burke et al.’s [23] findings on generational stigma. Ellis et al. [26] found that some participants identified facing self-denial in addition to self-stigma and acknowledged that whilst mental health difficulties were not a sign of weakness, they felt it was when it related to them personally. One young man stated, for example “…I guess there’s still a stigma of mental health being a weakness… I realise that it’s not but it’s just something that I’d find difficult coming to terms with” [26] (p. 6).

### Theme two: limited availability of information about mental health and help-seeking

#### Papers with this theme: 6 [24, 28–30, 32, 33]

*Limited availability of information* about common mental health disorders, especially anxiety, was also found to be a barrier to help-seeking amongst adolescent boys and young men. Within Meechan et al.’s [30] paper, some participants discussed how they view mental health as a disability and as something that cannot be recovered from, suggesting that they were not well-informed about the causes of or treatments for mental health problems. Clark et al. [24] identified a similar theme, where participants felt that they and their peers did not have the understanding that anxiety was a condition that could be treated or did not know how effective anxiety treatment would be for them. A perception of anxiety as “not a real illness” was also discussed [24] (p. 230). Further to this, it was found by Clark et al. [24] that participants held the belief that parents and teachers would also lack knowledge of mental health conditions such as anxiety, and therefore would not be able to offer help or support to adolescent boys and young men. Likewise, a lack of knowing where to go to get help served as a barrier, as well as fear of being prescribed medication from a health professional if they were to divulge mental health concerns [24, 29].

Sagar-Ouriaghli et al. [32] reported participants identified difficulty in assessing the severity of symptoms for anxiety and/or depression, and subsequently, when these would require professional intervention. This is reflected by one participant who shared that for them “the difficult part was thinking, convincing myself I need help” [32] (p. 7). Having limited awareness of common mental health conditions, and not understanding information when it was available, was also acknowledged by participants across several studies [28, 32, 33].

Some participants reported that limited mental health information meant they saw mental health conditions as ‘secret’ problems. In Clark et al. [24], participants considered mental health to be a hidden concern, while in Meechan et al. [30], mental health was seen as something that adolescent boys and young men could hide from others.

### Theme three: “male-friendly” campaigns to promote interventions or mental health literacy

#### Papers with this theme: 5 [23, 24, 29, 30, 32]

Creating *‘male-friendly’ mental-health campaigns and promoting literacy* was identified as a key facilitator for adolescent boys and young men amongst five studies, as a way of increasing the availability of information on mental health [23, 24, 29, 30, 32]. Expanding on the previous theme, using ‘simple’ or lay terms to explain mental health conditions can be seen as a facilitator to help-seeking. Additionally, transparency about psychological information and general service information was a facilitator to help-seeking. Clark et al. [24] highlight how mental health literacy campaigns could explain more clearly the severity of anxiety but also the treatability of the condition. Interestingly, some participants identified the importance of not emphasising mental health related words in interventions or support, with a focus group participant in one study stating “if you’re struggling with depression and what not as a man, let’s be real are you going to go to this workshop talking about men’s mental health? Probably not” [32] (p. 8).

Lynch et al. [29] identified the need for young men to be involved in creating strategies or interventions for mental health support. The term ‘mental health’ needs to be reframed (they suggest ‘mental fitness’) due to the negative connotations associated with the former [29]. Participants saw it as possible to “incorporate professional help seeking into masculine ideals” by creating a new meaning of the term for men [29] (p. 144).

### Theme four: help-seeking preferences

Another notable theme found, which was not related to barriers or facilitators, was the *help-seeking preferences* of adolescent boys and young men. Within this, two subthemes were identified: *informal vs. formal help-seeking* and *online vs. offline help-seeking.*

### Subtheme one: informal vs. formal help-seeking

#### Papers with this theme: 6 [22, 23, 26, 29, 30, 33]

There were many crossovers between the concepts that influence formal or informal routes to help-seeking, and online or offline help-seeking. The sources of offline support adolescent boys and young men turned to in Burke et al. [23] tended to be informal sources (i.e., not mental health professionals), such as family, friends, and teachers, as they found this support to be easier to access. In Ellis et al. [26], 86.6% of participants responded that they would recommend friends as a source of help. Adolescents and young men in Burke et al. [23] discussed how they had a preference for help-seeking from friends rather than family, due to familiarity, as well as the shared context of age and environment. However, for many adolescent boys and young men, CMN meant that they were often apprehensive about approaching their friends as they could not be sure they would receive appropriate, non-judgmental support [23, 26]. Participants in van der Schyff et al. [33] shared how they had low confidence in being able to cope with the reactions of friends and family if they did share their mental health concerns, which is similar to Lynch et al.’s [29] findings, where one participant shared that “I’ve tried talking before and I’ve been taken aback by the [negative] responses I’ve got” [29] (p. 142).

Concerns were also expressed by adolescent boys and young men over the fact that they could not keep anonymity when disclosing to friends or may be judged for showing their emotions [22, 30]. Participants in Meechan et al. [30] discussed the intersection of being a young, Black man in the UK, where community attitudes could make them less likely to seek help from their family or peers about mental health difficulties.

Formal help-seeking from mental health professionals was often seen as a more trustworthy pathway [22]. However, not all adolescent boys and young men shared this view. In Ellis et al. [26], participants reported a negative view towards counsellors and psychologists, and did not believe that they would really be able to help them with their problems. Others expressed that they worried about confidentiality when seeking treatment and were unsure whether they could trust mental health professionals [33]. Some saw formal support as something of a last resort; one participant, for example, in Meechan et al. [30] stated that “it would have to get really serious for [them] to get help” [30] (p. 6). Participants also saw formal support as something that existed within a system that was unkind and often discriminatory, especially towards Black men, and therefore was not something they felt they could access [30]. Additionally, professionals felt harder to approach for some as they were perceived as being older, majority white, and middle-class, and therefore not representative or relatable to most young men [26], and especially not to Black adolescent boys and young men in the UK [30]. Concerns surrounding how professionals would respond to different identities was also seen as a barrier to seeking formal support by adolescent boys and young men, particularly in Ireland [29]. Some young men were fearful of homophobic responses from professionals and felt that they could not be open about their identity when seeking help or treatment, which then made them more reluctant to seek the help altogether [29]. Practical barriers to seeking professional help (e.g., location of services, finding the time to access them, and access costs in some countries) were also mentioned by participants [26, 33].

### Subtheme two: online vs. offline help-seeking

#### Papers with this theme: 4 [22, 23, 26, 28]

There was an important distinction found between online and offline help-seeking for adolescent boys and young men. Young men were more likely to access online support, via search engines, and preferred this to other methods, often for reasons linked to the barriers discussed above [22, 26]. Participants saw this as a way of being independent and solving their own problems [26]. The tendency to turn to search engines and online support first was seen as a positive, as it could help to facilitate help-seeking by signposting adolescent boys and young men towards more professional services. For example, a participant in Ellis et al. [26] stated “I’d prefer to talk to someone on the Internet and then maybe make my way to a counsellor or a psychiatrist” [26] (p. 7). However Best et al. [22] note that not all the information online would be accurate or of high-quality, so it is important that this age group are taught how to filter out the good from the poor.

Seeking help offline was often closely linked to the relationships adolescent boys and young men had with their family and friends. Burke et al. [23] found that male participants who described themselves as being closer to their family trusted that they would understand their concerns and provide them with support, which therefore facilitated help-seeking. Conversely, some participants reported that this closeness would make them more hesitant about seeking help from family due to concerns over not wanting to trouble their parents, or wanting to show independence [23]. The latter concept was also reported by Liddle et al. [28], in which some adolescent boys and young men reported they would be less likely to seek help from their parents as they would get more embarrassed as they get older. In fact, those who responded to Liddle et al. [28] that they would be least likely to seek help from parents and family were found to be most at risk of mental health difficulties, according to the measures employed in their study.

Another source of offline help-seeking identified by Burke et al. [23] was teachers. Many of the same concepts of familiarity applied when adolescent boys and young men were considering whether to seek help from teachers, as they felt they knew staff and could trust them, but also there was an element of emotional distance that lessened embarrassment [23]. For example, one student described how they saw teachers and school staff as “that bridge between good help and trust” [23] (p. 4). Teachers were therefore often considered a valuable source of offline support.

### Theme five: self-compassion

#### Paper with this theme: 1 [27]

Heath et al. [27] was the only study that reported on the role of self-compassion in help-seeking among young men. Heath et al. [27] found that higher levels of self-compassion (showing kindness to oneself) were associated with fewer perceived barriers to help-seeking, such as self-stigma and resistance to self-disclosure due to the fear of consequences [27]. Higher levels of self-compassion also buffered the relationship between overall masculine norm adherence and self-stigma and self-disclosure concerns [27]. Undergraduate men who have higher levels of self-compassion may therefore be able to seek help without feeling shame or the pressure to adhere to masculine norms, by viewing help-seeking as a normal part of life [27].

## Discussion

This systematic review aimed to examine the barriers and facilitators to help-seeking for affective mental health issues in adolescent boys and young men. Twelve studies were included, with a relatively high risk of bias. The findings shed light on several important factors that influence help-seeking behaviours and highlight the need for targeted interventions and support strategies for this group. Overall, the review emphasises the complexity of help-seeking in this population, the powerful effect of masculine norms, and the significance of understanding factors that may facilitate engagement of young men.

To help clarify ideas about the barriers and facilitators, the identified help-seeking factors are discussed below in relation to two broad categories: *attitudes towards help-seeking* and *delivery of interventions.* Directions for future research and implications for practice are also presented.

### Attitudes towards help-seeking: the impact of social norms, including CMN and self-stigma

One key finding of this review is the strong impact of social norms on help-seeking behaviours, with CMN and self-stigma consistently recognised throughout the literature as interlinked barriers to adolescent boys and young men's help-seeking. The adherence to masculine ideals, such as self-reliance and emotional stoicism, was identified as part of this. The pressure to conform to these norms may lead to self-stigma, where young men perceive seeking help as a sign of weakness or a threat to their masculinity. These findings are supported by both Radez et al. [37] and Rickwood et al. [38] who outline how perceived gender roles influence help-seeking behaviours amongst adolescent boys and young men.

This highlights the need to challenge and redefine societal expectations of masculinity to create a more supportive environment that encourages help-seeking without undermining young men’s sense of masculinity. Adolescent boys and young men’s mental health help-seeking needs to be understood in the context of gender roles and negative and positive masculine stereotypes, and until gender stereotypes improve, interventions should focus on increasing the cultural acceptability of help-seeking for young men. Normalising anxiety and depression, specifically amongst adolescent boys and young men, and teaching topics such as GRC and compulsory masculinity, could increase help-seeking amongst this population. Future research should therefore explore innovative approaches to promoting help-seeking that actively challenge these norms and engage adolescent boys and young men in redefining what it means to be masculine in the context of mental health [39].

As mentioned above, the identified subthemes of CMN and self-stigma appear to be closely related. Self-stigma appears to result from CMN, with young men feeling the need to adhere to masculine stereotypes such as mental strength. In turn, this impacts their ability to seek help for mental health conditions, with studies reporting young men experience embarrassment and shame around asking for help [40]. However, the present review indicates that self-stigma should not be viewed as a monolithic factor which affects all adolescent boys and young men in the same manner. As reported in Meechan et al. [30], CMN and, by proxy, self-stigma, presents differently in Black communities compared to white communities. This may suggest that both conformity to masculinity and self-stigma should be understood within the context of intersectionality, as it is important to acknowledge and understand how racial, ethnic and cultural experiences may impact these factors [41]. This is supported by both Opara et al. [42] and Eylem et al. [43], stressing the need for mental health treatments to be culturally informed and understanding of the multifaceted experience of Black adolescent boys and young men. Mental strength was identified as a key traditional trait in Black UK communities and this needs to be understood for future research and interventions.

Self-stigma also appears to be passed down through generations of men [23, 30]. Therefore, future research could focus on identifying this pattern of generational stigma and how to break the cycle. Bosco et al. [44] found that there was a significant link between internalised stigma of help-seeking and disempowerment for adolescent boys and young men. They suggest that future interventions and research should focus more specifically on ‘gender-based prevention’. Interventions may have greater reach if they acknowledge the impact that gender stereotypes and self-stigma can have on adolescent boys and young men. There is a note of caution however, as Hayes et al. [45] discuss how targeted interventions, focusing on specific mental health conditions, may actually increase self-stigma and public stigma for individuals who are seeking help. Therefore, it is important to acknowledge the potentially polarising nature of targeted interventions when developing new programmes.

### Attitudes towards help-seeking: self-compassion

An interesting emerging finding from this review was the importance of self-compassion in adolescent boys and young men’s help-seeking, in that it buffered the relationship between adherence to masculine norms and other barriers to help-seeking such as self-stigma and risk associated with disclosure of mental health [27]. The impact of this was only referenced by one of the included studies in this review, which reflects a gap in wider research in this topic. The role of self-compassion in help-seeking seems to be a relatively new finding and could be a novel approach to identifying facilitators for adolescent boys and young men. Future research or interventions could focus specifically on the relationship between young men and self-compassion and how to increase the level of self-compassion within this group, to reduce the impact of barriers to help-seeking such as CMN and self-stigma. Neff and McGehee [46] examined the link between self-compassion and self-reliance for adolescents and young adults, finding that self-compassion was a good indicator of mental health. Adolescents and young adults with higher levels of self-compassion reported lower levels of both anxiety and depression. These findings broadly support Heath et al.’s [27] paper and could indicate a new avenue for research into mental health conditions amongst adolescents and young adults.

Although not widely researched, Compassion Focused Therapy (CFT) could potentially reduce self-stigma, thus reducing the impact of this as a barrier. CFT aims to help individuals identify and cope with difficulties, with a specific focus on treating oneself with kindness and compassion [47]. Despite there being limited research in this field, Luoma and Platt [48] note two studies which focused on the role of self-compassion in people with substance abuse issues and found that increasing self-compassion amongst participants resulted in reduced internalised shame and stigma. Evidently, there needs to be more research on the role of self-compassion and its impact on the mental health of adolescent boys and young men. There is currently one study in progress which is assessing the efficacy of CFT for reducing self-stigma of mental disorders [49], which could be important in showing the impact of CFT on self-stigma.

### Delivery of interventions: male-friendly campaigns to improve mental health literacy in adolescents and young adults

A clear barrier for adolescent boys and young men’s help-seeking is a lack of information about common mental health conditions, such as anxiety and depression, with the present findings generally reflecting the idea that young men either did not know how to recognise the symptoms of conditions or, if they did, did not know where to go for support. This echoes previous findings by Radez et al. [50], in which adolescents discussed the struggle they felt in recognising and understanding symptoms of depression and anxiety. The adolescents here expressed a need for more information and opportunities to learn about the symptoms and impacts of anxiety and depression, which could lead to better help-seeking behaviours, such as identifying when mental health is affecting one's life. Rickwood et al. [38] mirrors these findings, noting that “boys often do not recognise psychological distress for what it is, and if they do, they deliberately attempt to deny it” [38] (p. 24). Similarly, a review by Gulliver et al. [10] reported that adolescents and young adults either struggle to identify symptoms of mental health or, if they do identify symptoms, they are reluctant to acknowledge that they may need help for them. These findings together with the present review suggest that more targeted interventions need to be provided for adolescent boys and young men in order to improve their mental health literacy, thereby increasing help-seeking behaviours, which is also a view shared by Radez et al. [50].

Awareness campaigns for this demographic could promote the many types of mental health interventions already being offered by services, with a common theme in this review being the need for a wide range of easily accessible information to be available for adolescent boys and young men [24, 29]. A previous review by Sagar-Ouriaghli et al. [16] put forward that written materials could be used for advertising services, instead of face-to-face promotion, as using a non-contact approach was seen as less intimidating to young men. The methods of promotion were not discussed in detail in the studies included in the current review. However, Lynch et al. [29] did propose that young men should be involved in the design of strategies and materials, whatever format they may take. This could be done through the use of patient and public involvement (PPI) groups to ensure the suitability of the strategies for adolescent boys and young men. The articles included in the present review tend to agree that any interventions should utilise male-friendly language (i.e., avoid stigmatising terminology) and provide an informal space for shared discussion [23, 29]. One setting suggested by Burke et al. [23] that could potentially provide this more relaxed environment for interventions is schools and colleges, as these spaces offer more familiarity and benefit from the unique closely linked structure of students, teachers, and parents, which could encourage more adolescent boys and young men to seek help. Schools have been a popular place for effective mental health wellbeing and literacy interventions in the past, as this usually provides the opportunity to reach as many young people as possible [51, 52]. New youth mental health interventions continue to be trialled in a school setting, including the DISCOVER workshop in the Brief Educational Workshops in Secondary Schools Trial (BESST) [53], and the AWARE study [54].

Several included articles [23, 26, 30–32] identified the use of positive male role models as another way of encouraging adolescent boys and young men to access help. These could be beneficial by facilitating conversations about mental health and helping to shift the notion that help-seeking is not a masculine trait. Sagar-Ouriaghli et al.’s [16] review also supports this idea. Although it was focused on older male groups, that review discussed how the use of popular public figures in mental health campaigns, who were open about their own experiences, may help to promote services and the wide range of available interventions. This idea could be applied to younger males.

### Delivery of interventions: help-seeking preferences, including informal vs. formal and online vs. offline help-seeking

In general, adolescent boys and young men in the current review preferred informal help-seeking, from either family or friends. Parents and family members remain a popular source of support, especially for younger adolescents, but as they get older, they are more likely to turn to friends [23, 28]. The shared contexts and experiences that one may have with friends, especially those of the same age, can facilitate help-seeking in some young men as they trust that they will be understood [23]. Despite the findings in this review, there is not a great depth of previous research into how likely adolescent boys and young men are to seek help from their friends, or what might encourage or discourage them from doing so, as the majority of studies focus on young people in general; Rickwood et al.’s [9] study, for example, is in line with the idea that young people see family and friends as an essential source of support.

Nevertheless, help-seeking from friends may create separate concerns for adolescents and young adults, as young men in this review showed that they worried about being judged by friends or their community for their mental health, or were not confident in their ability to cope with their friends’ reactions to them seeking help [22, 23, 30, 33]. This presents a contradiction, as although adolescent boys and young men appear to be more likely to seek help from friends than any other source, they can be reluctant to take steps to do so, meaning that many may instead choose to deal with their problems alone rather than encountering the perceived risks of disclosure. This suggests that more needs to be done to encourage young men to seek support from their immediate social network as an initial route to help-seeking. Liddle et al. [28] proposes that interventions could target informal help sources by training them to provide appropriate support and information to improve help-seeking in young men. This is also supported by Rickwood et al. [9], who suggested that parents and peers could help to facilitate the help-seeking process for adolescents and young adults by helping them to identify problems and access further support. A focus within these interventions on addressing the wider attitudes of adolescent boys and young men, in a non-judgmental environment, may in turn improve discussion around mental health amongst young men, as they know that their peers share the same level of understanding. Interventions to promote help-seeking in this way have been trialled before, with one example of a successful strategy being the Silence is Deadly programme in Australia, which was found to be effective in increasing the likelihood of adolescent boys seeking help from their friends [55].

Formal help-seeking from professionals was mostly seen as a last resort for the most serious problems, and many males shared concerns that professionals were not relatable or approachable [26, 29, 30]. This is a concerning finding as it suggests that young men are unlikely to seek professional mental health support until their problems have developed into serious concerns requiring more specialist intervention. A review by Radez et al. [37] echoes this, finding that young people in general worried about being able to trust mental health professionals. The present review suggests that services need to do more to appeal to adolescent boys and young men and gain their trust, for example through the recruitment of male professionals who are more representative of minority groups, as this may encourage more young men to seek formal help [29, 30]. This finding is consistent with previous research; Bains et al. [56] previously discussed how the availability of professionals who were representative or understood the needs of minority groups was important for adolescent boys and young men. Ensuring young men are aware of the confidentiality and accessibility of services is frequently highlighted as a facilitator of professional help-seeking as this may help them to see formal support as a trustworthy option [57, 58], which relates to the above theme of creating male-friendly campaigns to promote services.

Adolescent boys and young men’s rather negative perceptions of seeking professional help, particularly face-to-face, may help to explain the preference shown for online help-seeking and related self-help strategies [22, 26]. This may suggest that, rather than encouraging young men to seek in-person support that they do not feel comfortable with, interventions to improve help-seeking could place a bigger emphasis on promoting good quality online methods of support. Indeed, it has been found in other work by Best et al. [59] that adolescent boys who did seek online help reported improved wellbeing. Similarly, offering interventions in online or computer-based formats may increase their acceptability to adolescent boys and young men. Ellis et al. [26] argues in favour of this and suggests that existing mental health services could aid the delivery and development of such interventions, which may be especially successful if they focus on active methods of behaviour change, rather than solely on increasing mental health knowledge. This finding is supported by Do et al. [60], who suggested that computer-based cognitive behavioural therapy (CBT) could be seen as a more accessible option, especially for adolescent boys and young men with low openness. However, CBT alone may not be sufficient in improving help-seeking attitudes, according to previous research [61]. Similarly, interventions to increase mental health literacy may improve attitudes towards help-seeking, but not actual help-seeking behaviour. One known approach that has been successful in improving both help-seeking attitudes and behaviour used a combination of factors including the use of self-referral, non-diagnostic language, and the offer of a brief CBT intervention [62]. Making improvements to mental health services in this way, and promoting them using a male-friendly approach, may therefore help to increase help-seeking in adolescent boys and young men by better addressing their needs and preferences.

### Implications for research and practice

The current review’s findings suggest several important directions for future research and practice. As outlined above, future research could particularly focus on the impact of self-compassion as a facilitator. There is a paucity of research on how race, ethnicity and sexuality may affect help-seeking in adolescent boys and young men, thus these factors could also provide topics for future research. Potential implications for future interventions to increase adolescent boys and young men’s help-seeking for mental health are also highlighted in this review, particularly regarding attitudes to help-seeking and to the delivery of interventions.

The evidence from the present review indicates that mental health services need to consider the impact of CMN and self-stigma, and also ensure cultural sensitivity when offering interventions. Clinical interventions and programmes to improve knowledge of mental health and help-seeking should be delivered with a male-friendly approach. Developing a stepped care approach for young men who may be initially reluctant, with signposting to services if they need further help, may also be helpful. More research is required into how interventions can be delivered in an effective way to all groups of young men. Whichever format and wherever these interventions are delivered, adolescent boys and young men should evidently be involved in their development from the start, to ensure they are suitable for the population they are being designed for.

This review has several strengths. This paper explores a wide range of literature concerning adolescent boys and young men’s patterns of help-seeking behaviour, which is especially important given the significant gap in research in this area. All articles were double screened by two independent authors, which increases the reliability of the review results. The present review has an explicit focus on affective mood disorders due to their commonality in adolescent boys and young men, however it is noted that this limited scope excludes mental health conditions which may have comorbidity with affective disorders, such as substance abuse disorders. Similarly, the review examines a specific and often overlooked period of adolescence, rather than the full spectrum of adolescence and emerging adulthood. Further reviews on help-seeking may wish to widen the inclusion criteria of disorders to reflect comorbid conditions, and to include the widest definition of adolescence. Future reviews may also benefit from examining non-Westernised samples and non-English language reviews, which could not be included in this review.

## Conclusion

Despite the significant lack of research into adolescent boys and young men’s help-seeking behaviour for affective mental health issues, this review has been able to identify several barriers and facilitators that could impact the decision to seek support. The results demonstrate the importance of considering the impact of CMN and self-stigma when designing future interventions to improve young men’s help-seeking behaviour. The review highlights self-compassion as a relatively new facilitator to adolescent boys and young men’s help-seeking, suggesting that future research should explore the potential benefits of incorporating this into therapeutic approaches, due to its potential positive impact on self-stigma. Finally, it is essential to involve young men in the creation of any new interventions or programmes to ensure that they are effective for their target audience, thereby increasing rates of help-seeking behaviour and lessening the impact of affective mental health issues on adolescent boys and young men.

## Supplementary Information

Below is the link to the electronic supplementary material.

Supplementary file1 (PDF 70 KB)

## Data Availability

No new data were created or analysed in the current study.
