# Supplementary material for: Why do young men not seek help for affective mental health issues? A systematic review of perceived barriers and facilitators among adolescent boys and young men
Source: Eur Child Adolesc Psychiatry. 2024 Jul 14;34(2):565–83. doi: 10.1007/s00787-024-02520-9 (PMC11868194; doi:10.1007/s00787-024-02520-9)
Supplement: Supplementary file 1 — Supplementary file1 (PDF 70 KB) [file 787_2024_2520_MOESM1_ESM.pdf]

Supplementary Information 1 for: **Why do young men not seek help for affective mental health issues? A systematic review of perceived barriers and facilitators among adolescent boys and young men**

Ayesha Sheikh[1&], Chloe Payne-Cook[2&], Stephen Lisk [3], Ben Carter[4], and June S. L. Brown[3*].
[&]Ayesha Sheikh and Chloe Payne-Cook contributed equally to this manuscript and share joint first authorship.

*Corresponding Author: Dr June S. L. Brown, june.brown@kcl.ac.uk; Institute of Psychiatry, Psychology and Neuroscience, King's College London, London, UK

An example of the search strategy used for the present review.

Example search strategy: PubMed

PubMed:

25/8/22

((((((((barrier[Title/Abstract]) OR (hurdle[Title/Abstract])) OR (obstruction[Title/Abstract])) OR (facilitator[Title/Abstract])) OR (support[Title/Abstract])) OR (encouragement[Title/Abstract])) OR (engagement[Title/Abstract])) OR (promotion[Title/Abstract])) AND ((((((((mental health[Title/Abstract]) OR (mental health disorder[Title/Abstract])) OR (anxiety[Title/Abstract])) OR (depression[Title/Abstract])) OR (stress[Title/Abstract])) OR (wellbeing[Title/Abstract])) OR (low mood[Title/Abstract])) OR (behavioural disorder[Title/Abstract])) OR (behavioral disorder[Title/Abstract]))) AND ((((help-seeking[Title/Abstract]) OR (treatment-seeking[Title/Abstract])) OR (help-seeking behaviour[Title/Abstract])) OR (help-seeking behavior[Title/Abstract])) Filters: Child: birth-18 years, Adolescent: 13-18 years, Adult: 19+ years, Adult: 19-44 years, Young Adult: 19-24 years

#1, , , Search: (((((((barrier[Title/Abstract]) OR (hurdle[Title/Abstract])) OR (obstruction[Title/Abstract])) OR (facilitator[Title/Abstract])) OR (support[Title/Abstract])) OR (encouragement[Title/Abstract])) OR (engagement[Title/Abstract])) OR (promotion[Title/Abstract]), 1,726,938

#2, , , Search: ((((((((mental health[Title/Abstract]) OR (mental health disorder[Title/Abstract])) OR (anxiety[Title/Abstract])) OR (depression[Title/Abstract])) OR (stress[Title/Abstract])) OR (wellbeing[Title/Abstract])) OR (low mood[Title/Abstract])) OR (behavioural disorder[Title/Abstract])) OR (behavioral disorder[Title/Abstract]), 1,575,536

#3, , , Search: (((help-seeking[Title/Abstract]) OR (treatment-seeking[Title/Abstract])) OR (help-seeking behaviour[Title/Abstract])) OR (help-seeking behavior[Title/Abstract]), 13,385

#4, , , Search: ((((((((barrier[Title/Abstract]) OR (hurdle[Title/Abstract])) OR (obstruction[Title/Abstract])) OR (facilitator[Title/Abstract])) OR (support[Title/Abstract])) OR (encouragement[Title/Abstract])) OR (engagement[Title/Abstract])) OR (promotion[Title/Abstract])) AND ((((((((mental health[Title/Abstract]) OR (mental health disorder[Title/Abstract])) OR (anxiety[Title/Abstract])) OR (depression[Title/Abstract])) OR (stress[Title/Abstract])) OR (wellbeing[Title/Abstract])) OR (low mood[Title/Abstract])) OR (behavioural disorder[Title/Abstract])) OR (behavioral disorder[Title/Abstract]))) AND ((((help-seeking[Title/Abstract]) OR (treatment-seeking[Title/Abstract])) OR (help-seeking behaviour[Title/Abstract])) OR (help-seeking behavior[Title/Abstract]))

#9, , , Search: ((((((((barrier[Title/Abstract]) OR (hurdle[Title/Abstract])) OR (obstruction[Title/Abstract])) OR (facilitator[Title/Abstract])) OR (support[Title/Abstract])) OR (encouragement[Title/Abstract])) OR (engagement[Title/Abstract])) OR (promotion[Title/Abstract])) AND (((((((((mental health[Title/Abstract]) OR (mental health disorder[Title/Abstract])) OR (anxiety[Title/Abstract])) OR (depression[Title/Abstract])) OR (stress[Title/Abstract])) OR (wellbeing[Title/Abstract])) OR (low mood[Title/Abstract])) OR (behavioural disorder[Title/Abstract])) OR (behavioral disorder[Title/Abstract]))) AND ((((help-seeking[Title/Abstract]) OR (treatment-seeking[Title/Abstract])) OR (help-seeking behaviour[Title/Abstract])) OR (help-seeking behavior[Title/Abstract])) Filters: Child: birth-18 years, Adolescent: 13-18 years, Adult: 19+ years, Adult: 19-44 years, Young Adult: 19-24 years
